# Benefits of resistant starch type 2 for patients with end-stage renal disease under maintenance hemodialysis: a systematic review and meta-analysis

**DOI:** 10.7150/ijms.51484

**Published:** 2021-01-01

**Authors:** Linpei Jia, Xingtong Dong, Xiaoxia Li, Rufu Jia, Hong-Liang Zhang

**Affiliations:** 1 Department of Nephrology, Xuanwu Hospital, Capital Medical University, Changchun Street 45#, 100053, Beijing, China.; 2 Central Hospital of Cangzhou, Xinhua Middle Street 201#, 061001, Cangzhou, Hebei Province, China.; 3 Department of Life Sciences, National Natural Science Foundation of China, Shuangqing Road 83#, 100085, Beijing, China.

**Keywords:** resistant starch type 2, hemodialysis, renal function, uremic toxin, proinflammatory response

## Abstract

**Background:** Resistant starch type 2 (RS2) has been documented to regulate gut microbiota and to improve the clinical outcomes of several diseases. However, whether RS2 may benefit patients with end-stage renal disease under maintenance hemodialysis (MHD) remains unknown.

**Methods:** We conducted a systemic review and meta-analysis of randomized controlled trials (RCTs). Adult patients receiving MHD were treated with RS2 (CRD42020160332). The primary outcomes were changes of uremic toxins, and the secondary outcomes were changes of inflammatory indicators, albumin and phosphorus.

**Results:** After screening 65 records, five RCTs (n = 179) were included. A significant decrease of blood urea nitrogen (weighted mean difference (WMD) = -6.91, 95% CI: -11.87 to -1.95, *I^2^* = 0%*, P* = 0.006), serum creatinine (WMD = -1.11, 95% CI: -2.18 to -0.05,* I^2^* = 44%, *P* = 0.04) and interleukin (IL)-6 in blood (standard mean difference (SMD) = -1.08, 95% CI: -1.64 to -0.53, *I^2^* = 35%, *P* = 0.0001) was revealed in the RS2 group. Analyses of blood levels of uric acid, p-cresyl sulfate, indoxyl sulfate, high sensitive C-reaction protein, albumin and phosphorus yielded no significant difference.

**Conclusions:** Our results suggest that RS2 may improve the residual renal function of patients under MHD and mitigate a proinflammatory response.

## Introduction

The growing prevalence of chronic kidney disease (CKD) threatens the public health, which leads to a high incidence of end-stage renal disease (ESRD) 1, 2. Maintenance hemodialysis (MHD) is a common method of renal replacement therapy for patients with ESRD. Although MHD may improve the life expectancies of patients 3, complications are increasingly recognized 4, such as metabolic syndrome, neurodegeneration and gut dysbiosis 5, 6.

Manipulation of gut microbiota is viewed as a promising approach to deal with a variety of diseases, such as neuropsychiatric disorders, CKD, and so forth 7. Due to an impaired ability of eliminating uremic toxins, specific uremic milieu of CKD impairs the intestinal barrier and promotes gastrointestinal inflammation, which in turn damages the microbial diversity in CKD patients 8-10. As kidney is a high-flow organ receiving one quarter of the whole blood volume, with limited anti-inflammatory or antioxidant ability 10, gut dysbiosis may aggravate the accumulation of uremic toxins in the body, whereby increasing micro-inflammation in the kidney 11. As a consequence, the increasingly augmented inflammation further damages renal function and forms a vicious circle.

Prebiotics are typically specialized nondigestible plant fiber compounds that circulate undigested through the upper gastrointestinal tract and enhance the activity of beneficial bacteria in the gut, presenting a favorable effect on the prognosis of CKD 12. Specific dietary supplements, including resistant starch (RS) 13, are frequently referred to as a modulator of gut flora, yet comprehensive insights into functional responses of microbiota to modulators are largely lacking. As a kind of special starch with high amylose and specific structures of amylopectin molecule 14, RS can escape the digestion in the small intestine and transit to colon, where it can be fermented by specialized members of bacteria 15. RS type 2 (RS2), in its natural granular form mainly in uncooked potato, corn, and green-banana flours, has been categorized as a prebiotics 16. In the colon, RS2 serves as a substrate to promote the growth of some beneficial bacteria, like *Bacteroidetes*, *Proteobacteria and so forth* 17. As a fermentable fiber, RS2 increases the production of short chain fatty acids (SCFAs), which reduce the permeability of intestinal walls and further inhibit renal microinflammation 17. High fiber dietary intake has been associated with a low risk of systemic inflammation and cardiovascular events in patients under MHD 18. A randomized controlled trial (RCT) in 56 patients under MHD showed that the levels of p-cresyl sulfate (PCS) and indoxyl sulfate (IS) were decreased after administration of RS2 for 6 weeks 19. Creatinine and inflammation biomarkers, including malondialdehyde, tumor necrosis factor-α and interleukin (IL)-6, were significantly decreased as well after RS2 treatment 20. Collectively, RS2 appears as a promising adjuvant therapy for patients under MHD. However, conclusions are inconsistent among studies on RS2 in CKD 19-23. Since no consensus for the clinical use of RS2 has been reached thus far, we herein evaluate the therapeutic effects of RS2 on MHD via an evidence-based method.

## Methods

To investigate the therapeutic effectiveness of RS2 on CKD, we searched for RCTs and analyzed changes of uremic toxins, inflammatory and other clinical indicators in eligible studies through a meta-analysis. Furthermore, we highlighted gaps in literature for guiding clinical studies in this area in the future. We conducted our meta-analysis according to the Preferred Reporting Items for Systematic Reviews and Meta-Analyses (PRISMA) statement (**Supplementary file 1**). The study has been registered at the International Prospective Register of Systematic Reviews (https://www.crd.york.ac.uk/PROSPERO/, No. CRD42020160332).

### Searching strategy

We combined entry terms of “chronic kidney disease”, “end stage renal disease”, “resistant starch” and synonyms of these terms to search articles in four major electronic databases, namely PubMed, EMBASE, Web of Science and Cochrane Library (see **Supplementary file 2** for the full search strategy). All articles published in the English language as of 20 October 2019 were searched without restrictions of origin of countries or article type. Internet-based information and conferences indexed in four electronic databases were also reviewed. Reference lists of all searched publications were screened to identify missing studies in the initial search by two independent researchers (XD and LJ).

### Study selection

Two researchers (XL and HLZ) assessed the initially yielded publications independently. Firstly, titles and abstracts were screened for appropriate studies. Then full-texts were assessed according to the inclusion criteria. Disagreement on eligibility was addressed by discussion and concluded after consensus. Patients under MHD refer to those with ESRD who had received hemodialysis for at least 3 months 24. The inclusion criteria were: (1) RCTs regardless of a design of blindness; (2) adult CKD patients receiving regular hemodialysis; (3) RS2 supplemented at any frequency and dosage. If the data of one cohort were published in several articles, the article with the longest therapeutic period and the largest sample size was selected. The exclusion criteria were: (1) non-MHD dependent CKD patients; (2) animal or *in vitro* experiments; (3) non-RCT clinical studies; (4) editorials, reviews, or publications without full-texts (i.e. conference abstracts); (5) studies using multiple supplements together as one intervention.

### Outcome measures

The major aim of our study was to evaluate the effect of RS2 on renal functions in patients with ESRD under MHD. Fasting blood samples were obtained in the morning before the hemodialysis session. The primary outcomes interdialysis changes of blood urea nitrogen (BUN), serum creatinine (Scr), uric acid (UA), PCS and IS. The secondary outcomes were changes of IL-6, high sensitive C-reaction protein (hsCRP), serum phosphorus and albumin. All indicators were measured in serum except that PCS and IS were measured either in serum or plasma.

### Data extraction

Characteristics of studies (researchers, publication year, design of blindness and sample size), demographic data of subjects (country, mean age and ratio of males), details of RS2 treatment (dosage, frequency and duration) and data of outcomes were extracted from each eligible trial by two researchers (XL and HLZ) independently. Discrepancies were judged by a third researcher (RJ) to ensure the accuracy.

### Risk of bias and quality assessment

The risk of bias for each selected study was estimated taking into consideration random sequence generation, allocation concealment, blinding of patients, blinding of outcome assessment, completeness of outcome data, selective reporting and other bias by the Cochrane Collaboration's tool for assessing the risk of bias 25. Quality of evidence was graded according to the risk of bias, inconsistency, indirectness, imprecision, and publication bias 26 with the Grading of Recommendation Assessment (GRADE) approach by the GRADEpro GDT 2015 (http://gradepro.org). Quality assessment and summary of findings (SoF) were performed by two independent researchers (LJ and XD). Disagreements were resolved with the help of a third researcher (HLZ) if necessary.

### Summary measures

Because all outcome data were continuous variables, data were synthesized by the inverse variance method and the random effects model. Variables were expressed as weighted mean difference (WMD). If variables were in different units or with great differences of measurements among included studies, standard mean difference (SMD) was used.

### Statistical analysis

We used the Review Manager (RevMan 5.3, The Nordic Cochrane Centre, Cochrane Collaboration, Copenhagen, Denmark) to perform the statistical analysis. *P* < 0.05 indicated a statistical significance. An inverse variance model was applied for continuous variables. We deployed a random effects model for a better accommodation of heterogeneity. A chi-square test on n-1 degrees of freedom was quantified for statistical heterogeneity 27. The Cochrane *I^2^* statistics were calculated to evaluate the heterogeneity among studies. Heterogeneity of data was considered to be acceptable if the *I^2^* value < 50% 28.

## Results

### Study selection and characteristics

**Figure 1:**
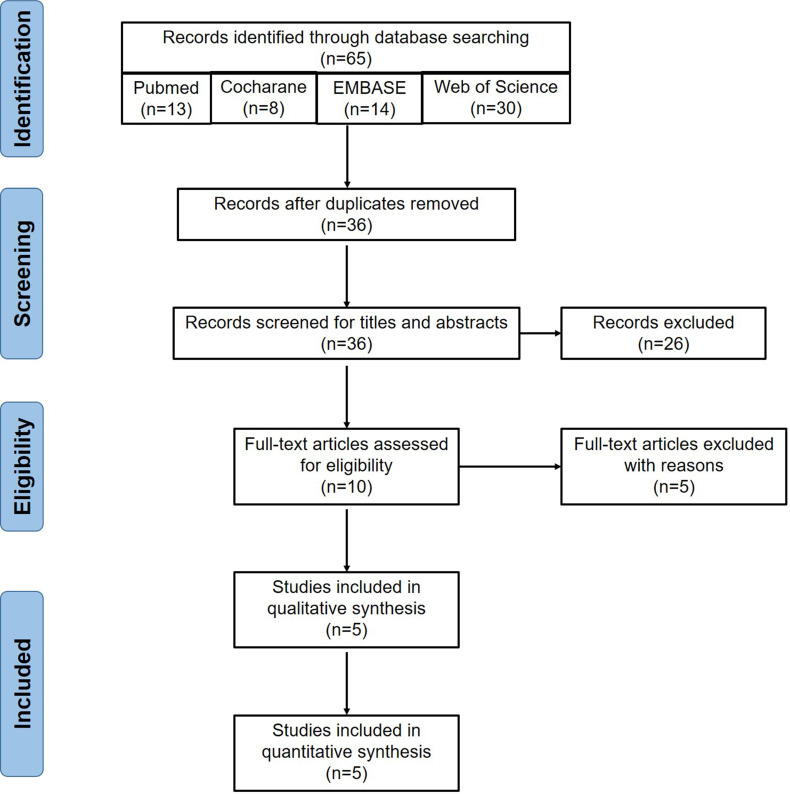
** Flow chart of eligible studies screening.** Initially, we searched 65 articles from the four databases, including 13 in PubMed, 14 in EMBASE, 30 in Web of Science and 8 in the Cochrane Library. Twenty-nine duplicates were removed. Then 26 publications were excluded after screening titles and abstracts, including 5 animal experiments, 13 non-chronic kidney disease studies, 5 non-resistant starch studies and 3 clinical trial registrations. Five articles were further excluded after reviewing the full-texts, including 3 reviews and 2 conference abstracts. Finally, 5 publications were included for the meta-analysis.

The detailed procedure of literature searching and selection is shown in **Figure 1**. A total of 65 publications were yielded after the initial searching. After removing 29 duplicates, we excluded an extra of 26 records via screening the titles and abstracts. Further, 5 records, including 2 conference abstracts and 3 reviews 17, 29, 30, were removed. Finally, 5 RCTs 19-23 including 179 patients under MHD were included after reviewing the full-texts (**Figure 1**). The summary of the included studies was shown in **Table 1**. Baseline characteristics of subjects in each study were summarized (**Table 2**). RS2 from two Ingredion companies (United States and Australia) was used for 4 weeks to 2 months.

### Risk of bias and SoF

**Figure 2:**
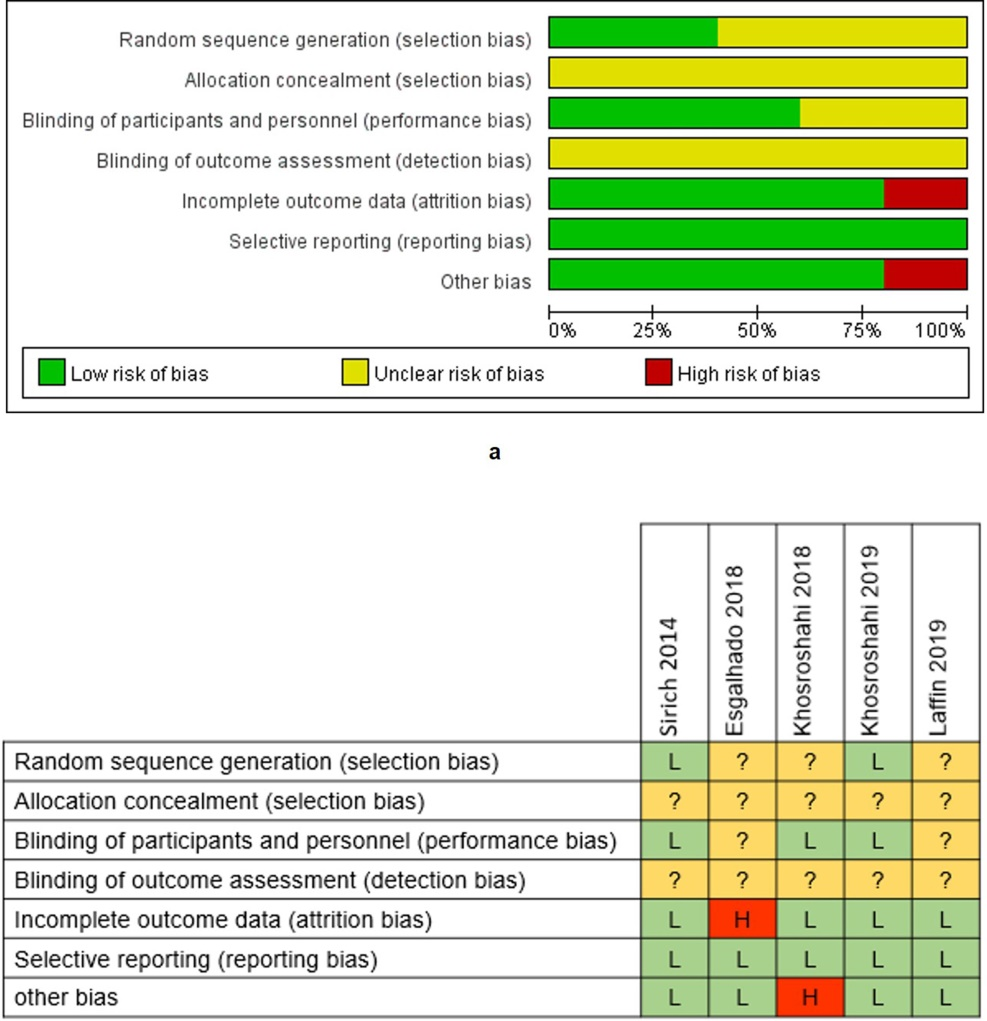
** Risk of bias graph and summary.** The low risk rate of random sequence generation was 40%, and low risk of blinding of patients was 60% (**a**). Incomplete outcome data and other bias had the low risk of 80% (**a**). The risk of allocation concealment and the blinding of outcome assessment were unclear (**a**). High risk appeared in incomplete outcome data and other bias with the rate of 20% (**a**). Esgalhado *et al.*'s study and Khosroshahi *et al.*'s study in 2018 was evaluated as high risks in one assessment. No study was estimated as low risk in all assessments (**b**).

We evaluated the risk of bias for each enrolled trial based on the Cochrane Collaboration's tool for assessing the risk of bias (**Supplementary file 3**). The allocation concealment and the blinding of outcome assessment were assessed as 100% unclear risk, while the reporting bias was assessed as 100% low risk (**Figure 2a**). Esgalhado* et al.*'s study and Khosroshahi *et al.'s* study, in 2018 respectively, were evaluated as high risk studies (**Figure 2b**), which might reduce the credibility of our study findings.

We then graded the quality of each outcome and presented the results as an SoF table (**Table 3**). Among all outcomes, IS and Scr were assessed as very low-quality because of the small sample size, inconsistent study results and the reporting bias. For outcomes assessed as very low-quality, we were uncertain about the estimates. PCS, BUN, UA, IL-6, hsCRP, albumin and phosphorus were graded as low-quality outcomes. For outcomes assessed as low-quality, we were uncertain about the estimates as well, and our estimates might be changed pending further investigations.

### Synthesis of results

#### Primary outcomes

**Figure 3:**
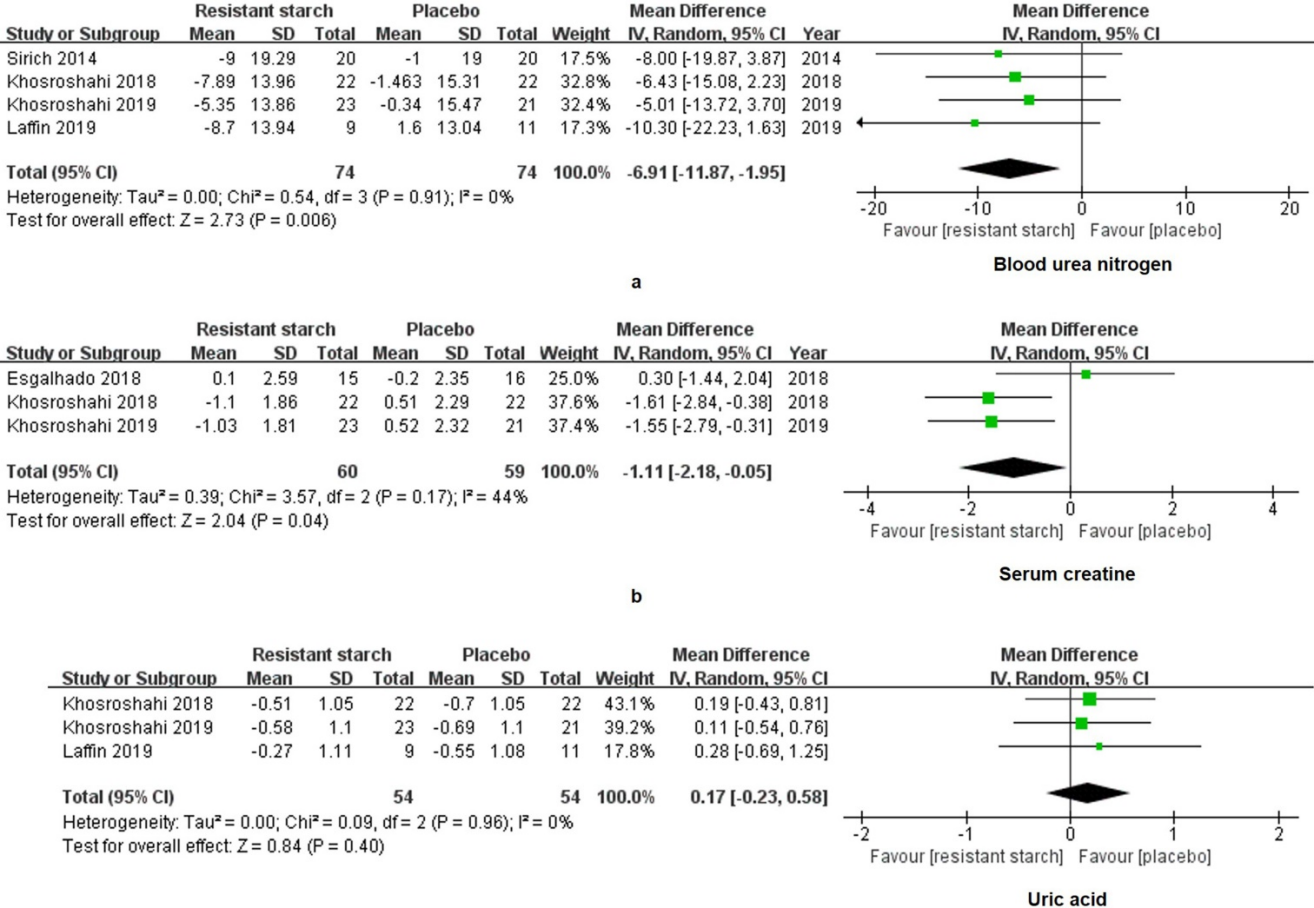
** Forest plots for comparisons of blood urea nitrogen (BUN), serum creatinine (Scr) and uric acid (UA).** Data of BUN (**a**) in four studies including 148 subjects were analyzed. A significant decrease of BUN was shown in the resistant starch type 2 (RS2) group compared with the control group (WMD = -6.91, 95% CI, -11.87 to -1.95, *I^2^* = 0%, *P* = 0.006). Scr (**b**) also showed a decrease in the RS2 group compared with the placebo in the meta-analysis (WMD = -7.43, 95% CI, -11.99 to -2.86, *I^2^* = 44%, *P* = 0.001). However, no significant change of UA (**c**) has been displayed after RS2 treatment (WMD = 0.17, 95% CI, -0.23 to 0.58, *I^2^* = 0%, *P* = 0.40).

Scr, BUN and UA are commonly used to evaluate the residual kidney function in the clinical practice. For the RS2 group, levels of BUN (WMD = -6.91, 95% CI: -11.87 to -1.95, *I^2^* = 0%, *P* = 0.006, **Figure 3a**) and Scr (WMD = -1.11, 95% CI: -2.18 to -0.05, *I^2^* = 44%, *P* = 0.04, **Figure 3b**) were significantly decreased as compared to the control group. The level of UA did not vary between the RS2 group and the control group (WMD = 0.17, 95% CI: -0.23 to 0.58, *I^2^* = 0%, *P* = 0.40, **Figure 3c**).

**Figure 4:**
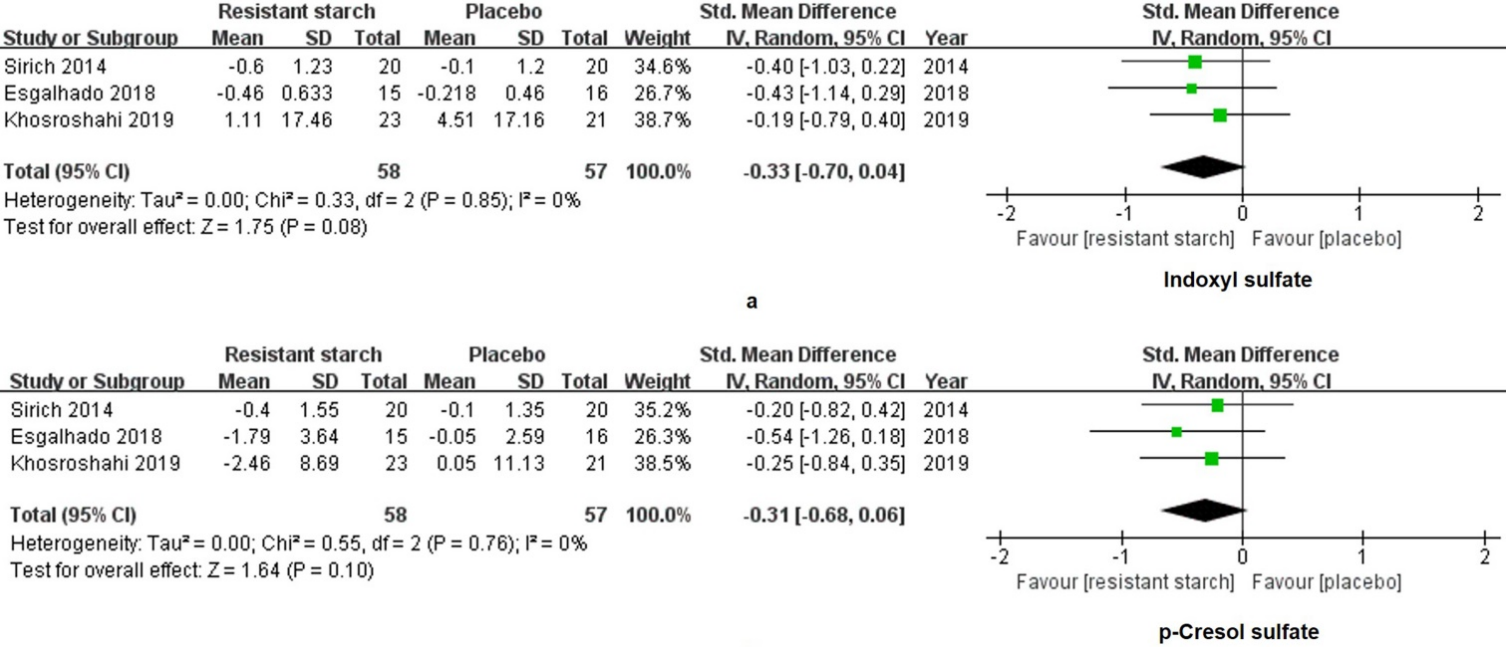
** Forest plots for comparisons of indoxyl sulfate (IS) and p-cresyl sulfate (PCS) in serum and plasma.** Sirich *et al.*'s, Esgalhado *et al.*'s and Khosroshahi 2019 *et al.*'s studies reported data of serum and plasma IS (**a**) and PCS (**b**) in resistant starch type 2 (RS2) and control patients under hemodialysis. Neither IS (SMD = -0.33, 95% CI, -0.70 to 0.04, *I^2^* = 0%, *P* = 0.08) nor PCS (SMD = -0.31, 95% CI, -0.68 to 0.06,* I^2^* = 0%, *P* = 0.10) was changed significantly after treatment with RS2.

We extracted and analyzed the data of 58 patients treated with RS2 and 57 patients treated with placebo. SMD was used to summarize measures for parameters with different units among studies. No changes were found in levels of IS (SMD = -0.33, 95% CI: -0.70 to 0.04, *I^2^* = 0%, *P* = 0.08, **Figure 4a**) and PCS (SMD = -0.31, 95% CI: -0.68 to 0.06, *I^2^* = 0%, *P* = 0.10, **Figure 4b**) of the RS2 group compared with the placebo group. Thus, RS2 could not decrease the levels of uremic toxins of patients under MHD.

#### Secondary outcomes

**Figure 5:**
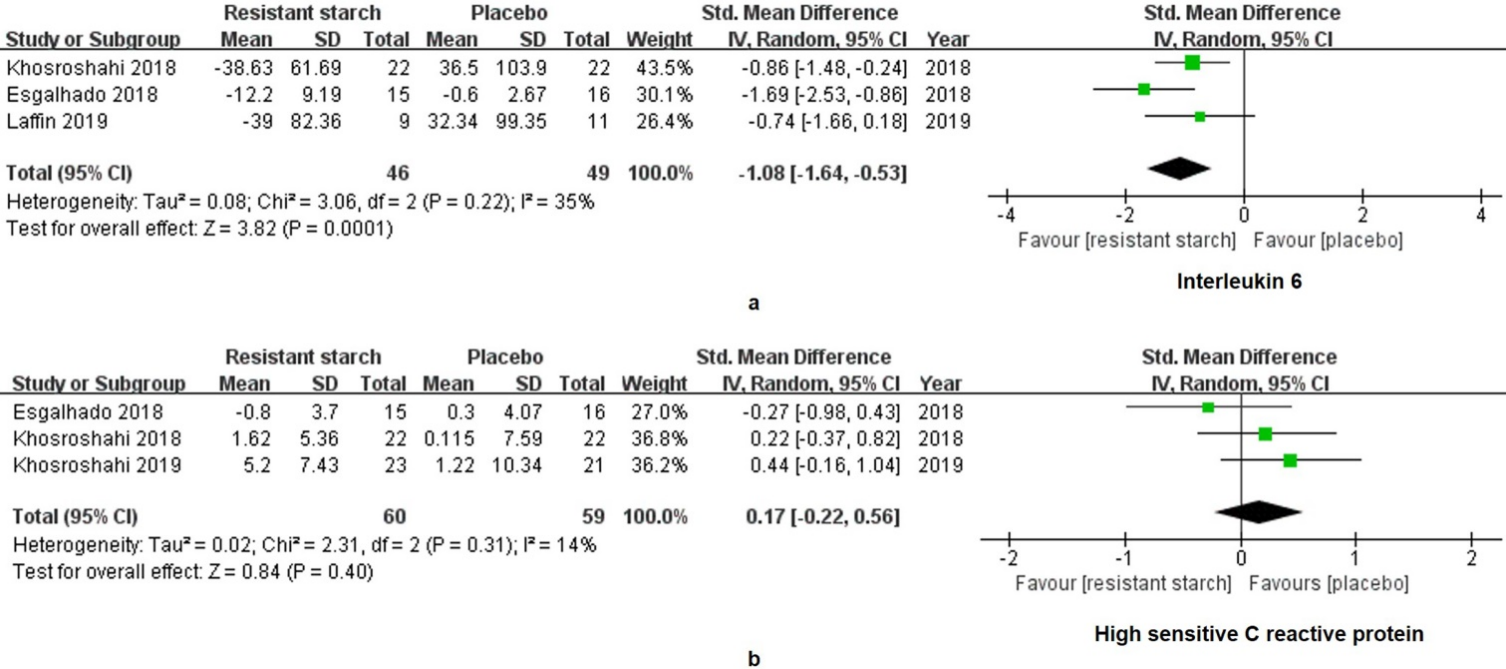
** Forest plots for comparisons of interleukin (IL)-6 and high sensitive C-reaction protein (hsCRP) in serum.** Three studies reported data of IL-6 in a total of 95 patients under maintenance hemodialysis (MHD). Resistant starch type 2 (RS2) could decrease the level of IL-6 in patients under MHD (SMD = -1.08, 95% CI: -1.64 to -0.53, *I^2^* = 35%, *P* = 0.0001, **a**). However, no significant change was found in serum level of hsCRP (SMD = 0.17, 95% CI: -0.22 to 0.56, *I^2^* = 14%, *P* = 0.40, **b**).

**Figure 6:**
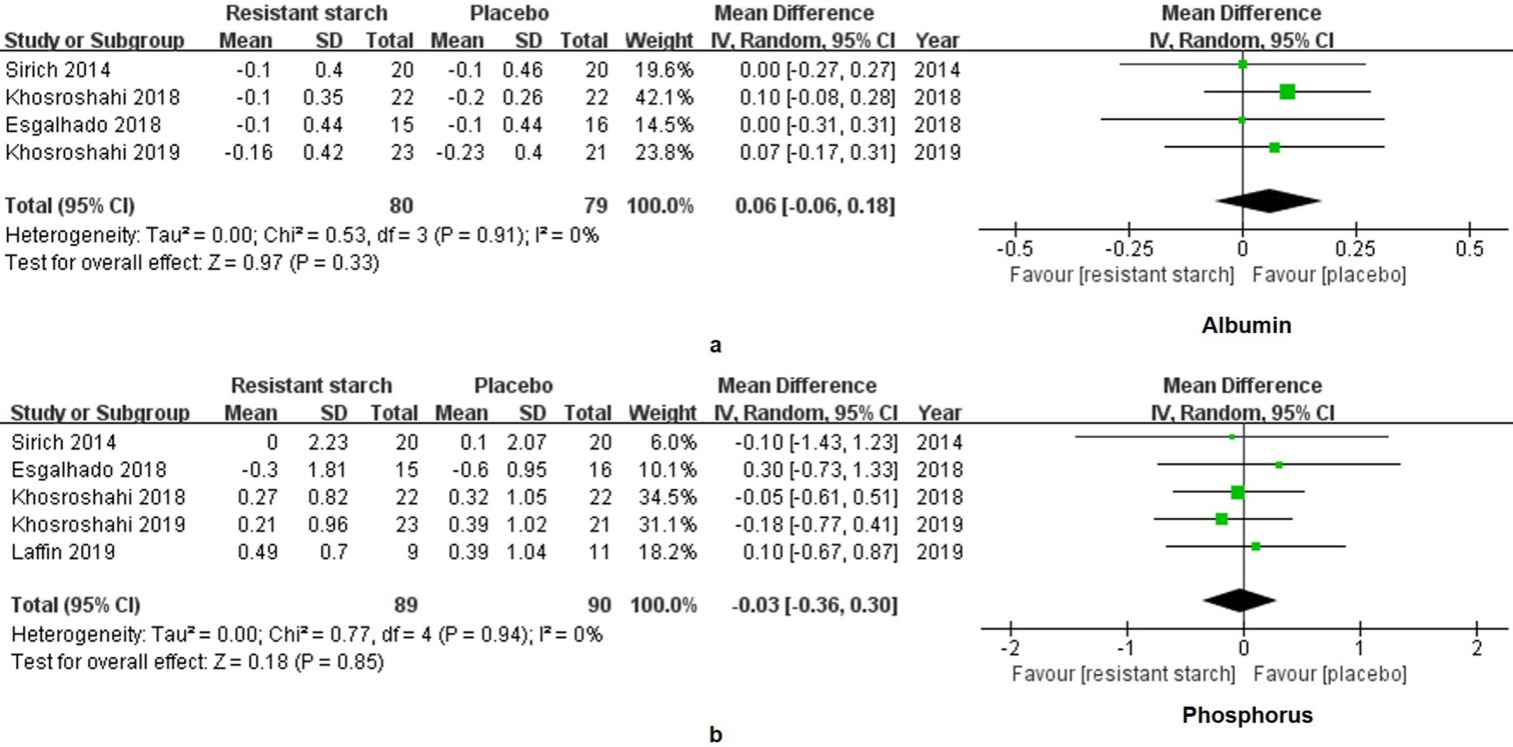
** Forest plots for comparisons of albumin and phosphorus in serum.** Four studies including 159 subjects reported the data of albumin (**a**). Levels of albumin were insignificantly altered in the resistant starch 2 group as compared with the control group (WMD = 0.06, 95% CI, -0.06 to -0.18, *I^2^* = 0%, *P* = 0.33). As for phosphorus (**b**), meta-analysis for five studies of 179 subjects showed insignificant change as well (WMD = -0.03, 95% CI, -0.36 to 0.30, *I^2^* = 0%, *P* = 0.84).

The levels of IL-6 in 95 patients under MHD were measured in 3 studies. A significant decrease of IL-6 was found in the treatment group (SMD = -1.08, 95% CI: -1.64 to -0.53, *I^2^* = 35%, *P* = 0.0001, **Figure 5a**). However, the change of hsCRP was insignificant (SMD = 0.17, 95% CI: -0.22 to 0.56, *I^2^* = 14%, *P* = 0.40, **Figure 5b**). Malnutrition and hyperphosphatemia are two major complications of MHD 31. Serum levels of albumin reflect the nutrition status of patients. We found insignificant changes of albumin in the RS2 group (WMD = 0.06, 95% CI: -0.06 to 0.18, *I^2^* = 0%, *P* = 0.33, **Figure 6a**). Neither was the level of phosphorus changed by the RS2 treatment (WMD = -0.03, 95% CI: -0.36 to 0.30, *I^2^* = 0%, *P* = 0.84, **Figure 6b**).

## Discussion

Research has rarely been focused on the effectiveness of RS2 in patients under MHD. In the current study, we summarized clinical trials and investigated the therapeutic effects of RS2 on patients under MHD, including changes of Scr, BUN, UA, PCS, IS, IL-6, hs-CRP, albumin and phosphorus. We found that RS2 could improve the residual kidney function of patients under MHD by decreasing levels of Scr in very-low certainty and reducing BUN and IL-6 in low certainty. We also found that the levels of PCS, IS, hs-CRP, albumin and phosphorus were not significantly altered by the treatment with RS2.

Patients under MHD are frequently complicated with chronic inflammation, which has a great impact on the early morbidity and mortality of patients 32. Chronic inflammation, together with medications, dietary changes *and so forth*, may result in alterations of the gut flora 33, 34. Gut dysbiosis results in an increased concentration of uremic toxins 35, leading to the inhibition of colonocyte proliferation, and even increasing DNA damage towards colonocytes 36, 37. The damaged gut barrier allows leakage of endotoxins into the circulation system 36, which facilitates the development of CKD by activating inflammatory cytokine production and aggravates chronic inflammation systemically and in kidney 38, 39. Presumably, RS2 may act as a prebiotic to modulate the proportion of gut microbiota by increasing the ratio of beneficial bacteria, such as *Lachnospiraceae*, *Ruminococcaea*e, *Faecalibacterium*, *etc* 23, 40. This may further reduce the production of uremic toxins so as to improve the integrity of the intestinal barrier and to decrease the micro-inflammation in other organs 41. Our meta-analysis showed that RS2 did benefit patients under MHD in terms of improving the residual kidney function; the reduced levels of Scr and BUN after RS2 treatment suggests that RS2 may prolong the interdialysis period. Younes *et al* previously reported that plasma urea concentrations were decreased both in normal and nephrectomized rats fed with fermentable carbohydrates, namely RS 42. Later, researchers confirmed the function of RS2 in reducing BUN, Scr and other CKD-related biomarkers in other animal experiments 40, 43. Our findings are consistent with previous bench studies 40, 43 and add knowledge to the benefit of RS2 for patients under MHD.

PCS and IS are two major uremic toxins, except for Src and BUN, excreted by the kidney, which are metabolites of bacterial products of p-cresol and indole respectively 44. PCS and IS are bound to plasma protein 45 and hence cannot be effectively eliminated by severely diseased kidneys or MHD 45. PCS and IS may accumulate in patients with CKD and further aggravate micro-inflammation in the kidney 46, 47. Several animal studies demonstrate that RS2 adjusted the proportion of intestinal symbiotic microbiota and increased the production of SCFAs, which could decrease the levels of PCS and IS 43. The effects of RS2 on PCS and IS were later corroborated by clinical trials 21, 22, 48. Wu *et al* demonstrated that dietary fiber intake could reduce the level of PCS in 203 CKD patients via a meta-analysis 48. Khosroshahi *et al* also revealed a significant decrease of PCS after RS2 treatment in patients under MHD 21. Instead, Esgalhado *et al* reported a totally opposite finding 22. In our meta-analysis of PCS and IS, neither of the two indicators was changed after treatment with RS2. Our results suggest that reduced levels of PCS and IS may not be the major mechanism underlying the suppressed inflammatory response after the treatment with RS2. Other factors or pathways may be implicated.

RS2 has been verified to reduce inflammatory biomarkers in various diseases 22. In the present study, a significant decrease of IL-6 was noted in patients under MHD upon treatment with RS2. Another meta-analysis also indicated that levels of inflammatory responses could be reduced by RS2 in patients with metabolic syndrome 49. Supplementation with RS2 to nephrectomized rats reduced nuclear factor kappa-B as well as proinflammatory molecules 50. We hereby postulate IL-6 as the key factor in the therapeutic effects of RS2. IL-6 is an important cytokine in the progression of CKD. In previous studies, increased levels of IL-6 were found in renal tissues of CKD patients and were related to risk of CKD complications 51, 52. Honda *et al* demonstrated that IL-6 was a reliable predictor of CKD related malnutrition and cardiovascular diseases 53. Kamińska *et al* suggested that the serum level of IL-6 was associated with coronary artery calcification and 5-year risk of all-cause mortality in CKD patients 54, 55. Importantly, renal cells, including podocytes, mesangial cells, endothelial cells *and so forth*, may express and secrete IL-6 51. In kidney, IL-6 could act as both a proinflammatory and an anti-inflammatory factor to promote cell proliferation, differentiation and tubulointerstitial fibrosis 56. In CKD, IL-6 is mainly increased in response to kidney injury to promote an inflammatory response 57. In specific, IL-6 activates signal transducer and activator of transcription 3 and increases the expression of fibroblast growth factor 23 in CKD 58, which further promotes the systemic inflammation and leads to the renal fibrosis 59, 60. In this regard, lowering the level of IL-6 in patients under MHD may alleviate disease process of ESRD. Since similar studies are limited, the detailed mechanism of IL-6 in RS2-treated patients under MHD merits further verification.

Hyperphosphatemia frequently complicates patients under MHD 61. Aggravated phosphorus burden in patients under MHD might be attributable to gut dysbiosis and consequent impaired activity of intracellular pathway of nicotinamide phosphoribosyltransferase and phosphate transporters in the intestines 61, 62. RS2 was shown to reduce the levels of phosphorus in animal studies 63. Indeed, RS2 might not directly participate in the metabolic pathway of phosphorus. The negative finding in the present study might be due to different levels of phosphorus intake, as the dietary recipe was not recorded in all of the included studies. This assumption may as well apply to albumin, an indicator of malnutrition; RS2 could neither increase nor reduce its serum levels.

When interpreting the results of this review, some limitations should be taken into account. First, the small sample size of the included studies may increase the reporting bias and undermine the evidence level. Second, because of insufficient data, effects of RS2 treatment on clinical endpoint events, death for instance, cardiovascular disease events *and so forth*, cannot be deciphered. Third, difference in duration and dosage of RS2 supplement may influence the efficacy in the meta-analysis; these discrepancies make it difficult to attain strong evidence for guiding clinical practice. Finally, all data were extracted from published articles. We did not contact authors for original data, which may render our meta-analysis subject to publication bias.

In conclusion, our results suggest that dietary supplement with RS2 may improve the residual renal function of patients under MHD by reducing inflammatory mediator IL-6. Nevertheless, results should be cautiously interpreted, because of the limited sample size and different treatment dosages. Large and pragmatic multicenter trials are thus necessary to corroborate the beneficial effects of RS2 supplementation on ESRD.

## Figures and Tables

**Table 1:** The summary of included studies for meta-analysis

Study	Country	Sample size	Formation of resistant starch type 2 (RS2) and placebo	Dosage	Duration of treatment
Sirich 2014 19	United States	20 patients and 20 controls	RS2: 15 g of high-amylose corn starch (Hi-maize 260), composed of approximately 40% digestible starch and 60% RS2;	1 sachet daily for 1 one week and 2 sachets daily for next weeks	6 weeks
			Placebo: 15 g of waxy corn starch.		
Esgalhado 2018 22	Brazil	15 patient and 16 controls	RS2: 16 g due to starch gelatinization for cookies;	1 sachet daily	4 weeks
			Placebo: 20 g manioc flour daily.		
Khosroshahi 2018 20	Iran	22 patients and 22 controls	RS2: 20 g or 25 g of 60% RS2 in HAM-RS2 enriched crackers;	1 sachet daily	8 weeks
			Placebo: regular wheat flour.		
Khosroshahi 2019 21	Iran	23 patients and 21 controls	RS2: 20 g or 25 g of 60% RS2 in HAM-RS2;	1 sachet daily	8 weeks
			Placebo: 20 g or 25 g of waxy corn starch.		
Laffin 2019 23	Iran	9 patients and 11 controls	RS2: 20 g or 25 g of 60% RS2 in HAM-RS2 enriched crackers;	1 sachet daily	2 months
			Placebo: regular wheat flour.		

**Table 2:** Baseline characteristics of study subjects

Study	Age, y		Male ratio, %		Body mass index, kg/m^2^		Duration of treatment, y	
	RS2	Control	RS2	Control	RS2	Control	RS2	Controls
Sirich 2014 19	54.0 ± 14.0	58.0 ± 13.0	55.0	65.0	29.0 ± 7.0	29.0 ± 6.0	5.0 (2.0, 6.0)	3.0 (2.0, 6.0)
Esgalhado 2018 22	56.0 ± 7.5	53.5 ± 11.5	46.7	68.8	26.2 ± 5.0	26.6 ± 5.3	4.2 ± 3.1	3.7 ± 2.2
Khosroshahi 2018 20	52.0 ± 11.0	60.0 ± 14.0	54.5	72.7	23.8 ± 2.4	23.3 ± 1.7	5.1 ± 1.3	4.8 ± 1.8
Khosroshahi 2019 21	53.2 ± 10.2	57.9 ± 13.3	56.0	60.0	24.4 ± 2.2	23.9 ± 1.6	5.1 ± 1.3	4.8 ± 1.8
Laffin 2019 23	53.8 ± 11.8	57.6 ± 9.0	66.7	63.6	NR	NR	NR	NR

Note: Data are presented as the mean ± SD or median (25^th^ percentile, 75^th^ percentile). RS2, resistant starch type 2; NR, not reported.

**Table 3:** Summary of findings of resistant starch type 2 (RS2) for end stage renal disease patients under maintenance hemodialysis

Patient or population: patients with chronic kidney disease; Intervention: RS2				
Outcomes	Illustrative comparative risks* (95% CI)		No. of Participants (studies)	Quality of the evidence (GRADE)
	Assumed risk	Corresponding risk		
	Control	RS2		
BUN		The mean BUN in the intervention groups was **6.91 mg/dL lower** (11.87 to 1.95 lower)	148 (4 studies)	⊕⊕⊝⊝ low^1,2^
Scr		The mean Scr in the intervention groups was **1.11 mg/dL lower** (2.18 to 0.05 lower)	119 (3 studies)	⊕⊝⊝⊝ very low^1,3,4^
UA		The mean UA in the intervention groups was **0.17 mg/dL higher** (0.23 lower to 0.58 higher)	108 (3 studies)	⊕⊕⊝⊝ low^1,5^
IS		The mean IS in the intervention groups was 0**.33 SD lower** (0.7 lower to 0.04 higher)	115 (3 studies)	⊕⊝⊝⊝ very low^1,3,6^
PCS		The mean PCS in the intervention groups was **0.31 SD lower** (0.68 lower to 0.06 higher)	115 (3 studies)	⊕⊕⊝⊝ low^1,3^
IL-6		The mean IL-6 in the intervention groups was **1.08 SD lower** (1.64 to 0.53 lower)	95 (3 studies)	⊕⊕⊝⊝ low^1,3^
hsCRP		The mean hsCRP in the intervention groups was **0.17 SD higher** (0.22 lower to 0.56 higher)	119 (3 studies)	⊕⊕⊝⊝ low^1,3^
Albumin		The mean albumin in the intervention groups was **0.06 g/dL higher** (0.06 lower to 0.18 higher)	159 (4 studies)	⊕⊕⊝⊝ low^1,3^
Phosphate		The mean phosphate in the intervention groups was **0.03 mg/dL lower** (0.36 lower to 0.30 higher)	179 (5 studies)	⊕⊕⊝⊝ low^1,3^

*The basis for the **assumed risk** (*i.e.* the median control group risk across studies) is provided in footnotes. The **corresponding risk** (and its 95% confidence interval) is based on the assumed risk in the comparison group and the **relative effect** of the intervention (and its 95% CI);**BUN:** blood urea nitrogen;** CI:** confidence interval; **hsCRP:** high sensitive C-reaction protein; **IL-6:** interleukin 6; **IS:** indoxyl sulfate; **PCS:** p-cresol sulfate; **RS2**: resistant starch type 2; **Scr:** serum creatinine; **SD:** standard deviation; **UA:** uric acid. GRADE Working Group grades of evidence:**High quality:** Further research is very unlikely to change our confidence in the estimate of effect;**Moderate quality:** Further research is likely to have an important impact on our confidence in the estimate of effect and may change the estimate;**Low quality:** Further research is very likely to have an important impact on our confidence in the estimate of effect and is likely to change the estimate;**Very low quality:** Very uncertain about the estimate. ^1^ Imprecise due to the small sample size (less than 300) in all studies. Thus, the evidence quality was down-graded as one level.^2^ One study was inconsistent with the other three in BUN, thus the quality of evidence was down-graded by one level.^3^ One study was supported by food company of RS2, thus the quality of evidence was down-graded by one level.^4^ One study was inconsistent with the other two in Scr, thus the quality of evidence was down-graded by one level.^5^ One study was inconsistent with the other two in UA, thus the quality of evidence was down-graded by one level.^6^ One study was inconsistent with the other two in IS, thus the quality of evidence was down-graded by one level.
